# Land Cover and Climate Change May Limit Invasiveness of *Rhododendron ponticum* in Wales

**DOI:** 10.3389/fpls.2018.00664

**Published:** 2018-05-18

**Authors:** Syed A. Manzoor, Geoffrey Griffiths, Kotaro Iizuka, Martin Lukac

**Affiliations:** ^1^School of Agriculture, Policy and Development, University of Reading, Reading, United Kingdom; ^2^Department of Geography and Environmental Sciences, University of Reading, Reading, United Kingdom; ^3^Center for Spatial Information Science, University of Tokyo, Tokyo, Japan; ^4^Faculty of Forestry and Wood Sciences, Czech University of Life Sciences Prague, Prague, Czechia

**Keywords:** climate change, invasive species, Maxent, Markov chain, multi-layer perceptron, species distribution modeling

## Abstract

Invasive plant species represent a serious threat to biodiversity precipitating a sustained global effort to eradicate or at least control the spread of this phenomenon. Current distribution ranges of many invasive species are likely to be modified in the future by land cover and climate change. Thus, invasion management can be made more effective by forecasting the potential spread of invasive species. *Rhododendron ponticum* (L.) is an aggressive invasive species which appears well suited to western areas of the UK. We made use of MAXENT modeling environment to develop a current distribution model and to assess the likely effects of land cover and climatic conditions (LCCs) on the future distribution of this species in the Snowdonia National park in Wales. Six global circulation models (GCMs) and two representative concentration pathways (RCPs), together with a land cover simulation for 2050 were used to investigate species' response to future environmental conditions. Having considered a range of environmental variables as predictors and carried out the AICc-based model selection, we find that under all LCCs considered in this study, the range of *R. ponticum* in Wales is likely to contract in the future. Land cover and topographic variables were found to be the most important predictors of the distribution of *R. ponticum*. This information, together with maps indicating future distribution trends will aid the development of mitigation practices to control *R. ponticum*.

## Introduction

Invasive alien species are considered the second biggest threat to global biodiversity, after habitat degradation (Gurevitch and Padilla, 2004; Powell et al., 2013). Invasive plant species alter the dynamics of plant communities and thus threaten the stability and functioning of established ecosystems by affecting nutrient cycles and net primary productivity, affecting soil health by increasing soil acidity, posing risk for pollinators, inhibiting regeneration of native species, and competing with native flora (Manchester and Bullock, 2000; Ehrenfeld, 2003; Snowdonia Rhododendron Partnership, 2015; Tiedeken and Stout, 2015). Plant invasion causes significant economic losses to crop and livestock farmers around the world (Peterson et al., 2008). Various studies estimate that the global monetary value of direct damage and associated control of invasives exceeds $100 billion per annum (Pimentel et al., 2005). However, since there are many invasive species with no recorded damage costs, the true figure is likely to be many times higher than these estimates (Bradshaw et al., 2016). Several studies have highlighted the potential impacts of global climate change on population dynamics of invasive species, with secondary effects on host plant communities and ecosystems (Chapin et al., 2000; Peterson et al., 2008). During the last century, global average temperatures have increased by 0.85°C above pre-industrial levels and are expected to further increase by 0.3–4.8°C by 2100 (IPCC, 2013). Changes in climatic conditions may render some regions more or less suitable for invasive plants thus increasing or decreasing their range (Bradley et al., 2010). Effects of climate change on invasiveness of alien species must be considered and any prediction of future distribution should include a range of climate change scenarios.

Once an invasive species has established itself, one of the most cost effective ways to reduce its threat is to map its current distribution and take pre-emptive measures to prevent further expansion (Alves et al., 2017). Such targeted management of biological invasions is not possible without information about the likely future distribution of invasive species. In this context, species distribution models (SDMs) present a workable opportunity to examine future changes in species distribution (Taylor et al., 2012). As climate is a strong determinant of habitat suitability of plant species (Marino et al., 2011), SDMs are often driven by environmental variables. Also known as ecological niche models, they are successfully being used for projecting the impacts of climate change on plant distributions (Elith et al., 2006; Ramírez-Albores et al., 2016). In principle, species are assumed to exist in a “niche” described by ecological requirements of the species. SDMs characterize these ecological space of a species and subsequently identify vulnerable locations based on the environmental suitability of the species (Trivedi et al., 2008).

In addition to climate, distribution of invasive plant species is often strongly linked to land cover type. For instance, transportation corridors, continuous grasslands, forest areas, and proximity to human settlements are often reported as strong determinants of species spread (Decker et al., 2012). A score of SDM studies indicates that land cover is often a far better predictor of species habitat suitability than climatic variables (Yang et al., 2013; Alkhamis et al., 2017; Bosso et al., 2017; Guo et al., 2017; Padalia and Bahuguna, 2017). Changes in land cover can affect both quality and quantity of suitable habitat, in some instances the landscape variables alone can accurately predict the distribution of a species (Hailu et al., 2017). It is therefore recommended to consider climate and land cover change in combination when exploring species' niche shifts in future (Dale, 2017). However, despite the fact that land cover is an integral part of species' ecological niche, the majority of SDM studies investigating species' future distribution ignore it and assume that species' future distribution is only driven by shifts in climatic variables (Khanum et al., 2013; Khadka and James, 2017; Qin et al., 2017). The history of climatic changes and human land use shows that land cover types will shift, any modeling of species' future distribution based merely on climatic variables may lead to a severely misleading prediction (de Chazal and Rounsevell, 2009).

In Europe, *Rhododendron ponticum* (L.) is an invasive plant species that was introduced to the United Kingdom in the eighteenth century as an ornamental plant. It is a perennial, evergreen shrub that generally invades woodlands (Tiedeken and Stout, 2015), although it has been shown to colonize other types of habitats too. The main ancestor is reported to be the population of *R. ponticum* resident at the southern tip of Spain. The successful invasion of *R. ponticum* in the UK is attributed to a range of its ecological and biological characteristics: it produces great amounts of seeds which are wind-dispersed, can tolerate shade and thus outcompetes flora under closed canopies and can easily colonize low-nutrient sites (Dehnen-Schmutz and Williamson, 2006). It often prevents germination of native plant species by casting a dense shade and by releasing toxins into the soil (Stephenson et al., 2006). Germination of *R. ponticum* seeds may occur on a number of substrates, including tree stumps and mosses covering bare ground (Cross, 1981) The UK invasion by this shrub has been more intense in Western and North Western parts, which are the comparatively cooler and wetter areas of Britain. A genetic analysis of the British population of *R. ponticum* has confirmed the presence of genes from *R. catawbiense* (Michx), suggesting past hybridization between the two species. *R. catawbiense* is a species native to North America and characterized by greater cold tolerance (Erfmeier et al., 2011; Snowdonia Rhododendron Partnership, 2015; Die et al., 2017), a trait that may increase invasiveness of *R. ponticum* in the UK. However, an in-depth analysis is still required to identify the other key environmental factors responsible for colonization and spread of this species. Of the various parts of U.K. invaded by *R. ponticum*, Wales is one of the worst affected regions. In this study, we focus on the Snowdonia National Park in Wales where *R. ponticum* is identified as a major invasive species affecting large areas of the National Park (Jackson, 2008) indicating that current environmental, topographic and land cover conditions in Snowdonia represent a range of conditions very suitable for *R. ponticum*.

We examine the current and future distribution of *R. ponticum* in Snowdonia National Park, Wales, UK under current and future land cover and climatic conditions (LCCs). Our modeling effort aims to, (a) delineate “invasion hotspots” for *R. ponticum* in Snowdonia National Park, (b) identify key ecological factors driving the spread of *R. ponticum* in the park, and (c) identify likely spatial patterns of habitat suitability under future climate conditions to establish a theoretical reference framework for management plans to combat the potential invasion of *R. ponticum*.

## Materials and methods

We used MAXENT, a maximum-entropy based machine learning algorithm to model the distribution *R. ponticum* (L.) in Snowdonia National Park. MAXENT predicts the probability distribution of a species on the basis of a given set of environmental variables and presence-only species occurrence data (Phillips et al., 2004). We selected MAXENT because, (a) it does not require absence data (Phillips et al., 2006), (b) it efficiently handles complex interactions between predictor and response variables (Elith et al., 2006), (c) being a generative model, it performs better than discriminative models when it comes to modeling with presence-only records and, (d) it can be run with both categorical and continuous data variables (Elith et al., 2011). There are several known limitations of the MAXENT modeling environment; (a) sensitivity to small sample size and questionable occurrence records (Elith et al., 2011), (b) use of overly complex models due to user over-reliance on default model calibration settings (Moreno-Amat et al., 2015), and (c) biased performance due to errors in sampling effort or spatial autocorrelation of occurrence records (Veloz, 2009). In this study, we countered these model limitations by; (a) using reasonably large sample size and applying recommended screening and verification of occurrence records, (b) tuning the model by identifying optimal model calibration settings, and (c) accounting for sampling bias and applying spatial filters to reduce spatial autocorrelation.

### Pre-processing of occurrence records and predictor variables

Presence-only occurrence records of *R. ponticum* were obtained from COFNOD (Local Environmental Records Centre in Wales, UK). A dataset totaling 436 occurrence records originating from a continuous field observation campaign spanning the period between 1981 and 2016. COFNOD has confirmed that the entire area of Snowdonia National Park had been thoroughly surveyed by ground surveys and remote sensing tools, thus minimizing the possibility of sampling bias in the dataset. Consequently, in our modeling effort we covered the entire area of the national park, generating 10,000 random background points to be selected from in each replicate run of the model. Spatial uncertainty of all occurrence records was verified and all duplicate or not geo-referenced occurrence points were removed. Occurrence data were spatially rarefied by eliminating all but one point present within a single grid cell of the predictor variable layers to reduce spatial autocorrelation. As a result, the number of occurrence points used for model calibration and verification was reduced from 452 to 92.

We considered a total of 23 predictor variables (Table 1) covering Snowdonia National Park at a cell resolution of 30–arc-seconds (~1 km, worldclim.org, version 1.4, Hijmans et al., 2005). These 23 variables were selected on the basis of published information on plant-habitat associations of *R. ponticum*. We included bioclimatic variables, together with a land cover variable as *R. ponticum* is a habitat-specialist and thus sensitive to land cover type. In addition, we included topographic factors such as slope, aspect and altitude as these factors are also known to limit the distribution of this species (Erfmeier and Bruelheide, 2004; Eşen et al., 2004; Stephenson et al., 2006; Harris et al., 2011). In all, our predictor dataset consisted of 19 climatic variables were complemented by 3 topographic and 1 land cover variable. A Digital Elevation Model (Shuttle Radar Topography Mission, https://lta.cr.usgs.gov/SRTM1Arc) with spatial resolution of 30 m was used to derive three topographic variables: altitude, aspect and slope. Land Cover data originates from “The European Space Agency CCI” global land cover product available at 300 m of spatial resolution (www.esa-landcover-cci.org). The whole set of 23 variables (19 climatic, 1 land cover, and 3 topographic) was re-sampled to 1 km spatial resolution and masked to the extent of Snowdonia National Park. A combination of expert knowledge, published studies on *R. ponticum* invasiveness in the UK and statistical methods was used to select an appropriate set of predictor variables to reduce the negative impact of multicollinearity and to conform to statistical assumptions (Syfert et al., 2013). We removed highly correlated variables by applying a Pearson correlation coefficient cutoff of *r* ≤ 0.85 to select the variable layers for use in final model runs (Graham, 2003).

**Table 1:** Predictor variables used in the study, variables highlighted in bold were selected to run all models presented in this study.

**Code**	**Predictor variable**	**Unit**
BIO 1	Annual mean temperature	°C
**BIO 2**	**Mean diurnal range [monthly (max temp – min temp)]**	°C
**BIO 3**	Isothermality (BIO2/BIO7)* **100**	
**BIO 4**	**Temperature seasonality (standard deviation** *100)	C of V
BIO 5	Max temperature of warmest month	°C
BIO 6	Min temperature of coldest mont	°C
BIO 7	Temperature annual range (BIO5-BIO6)	°C
BIO 8	Mean temperature of wettest quarter	°C
**BIO 9**	**Mean temperature of driest quarter**	°C
BIO 10	Mean temperature of warmest quarter	°C
BIO 11	Mean temperature of coldest quarter	°C
BIO 12	Annual precipitation	mm
BIO 13	Precipitation of wettest month	mm
BIO 14	Precipitation of driest month	mm
**BIO 15**	**Precipitation seasonality (coefficient of variation)**	C of V
BIO 16	Precipitation of wettest quarter	mm
BIO 17	Precipitation of driest quarter	mm
BIO 18	Precipitation of warmest quarter	mm
BIO 19	Precipitation of coldest quarter	mm
**Altitude**	**Altitude**	m
**Aspect**	**Aspect**	°
**Slope**	**Slope**	°
**Land cover**	**Land cover**	

### Habitat suitability under climate and land cover change scenarios

Projected future climatic conditions for the year 2050 based on the IPCC 5th assessment report were used to assess the potential effects of climate change on *R. ponticum* habitat suitability in Snowdonia National Park. We used the following six GCMs projections: BCC-CSM1-1, CCSM4, GISS-E2-R, MIROC5, HadGEM2-ES, and MPI-ESM-LR. These are some of the most recent GCMs, also used in the Fifth Assessment IPCC report and are currently considered the most reliable GCMs for future climate projections (IPCC, 2014). The assessment was made under two Representative Concentration Pathways: RCP 4.5 and RCP 8.5. RCP 4.5 describes a scenario where GHG emissions are stabilized and thus represents a stable scenario, while RCP 8.5 is a scenario depicting an extreme situation where GHG emissions increase until 2100 (Akhter et al., 2017).

**Figure 1:**
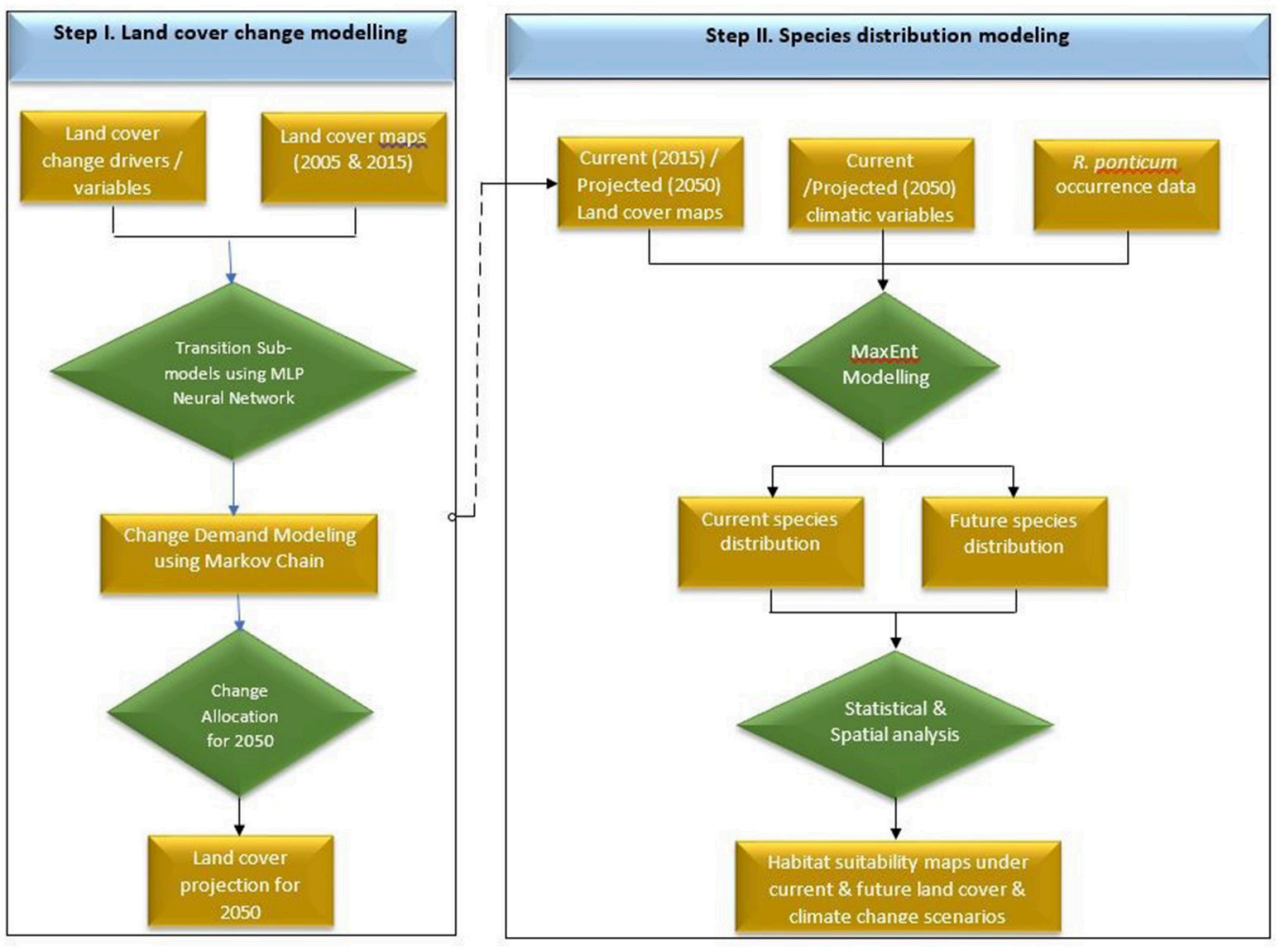
Flow chart detailing sequential steps carried out in land cover simulation (Step I) and Maxent based species distribution modeling (Step II) of *R. ponticum* in Snowdonia National Park, Wales.

Land cover for 2050 was simulated in Terrset software (Eastman, 2006) using recommended protocols (Dadhich and Hanaoka, 2010; Ozturk, 2015; Ye et al., 2018). Making use of the Multi-layer Perceptron-Markov Chain (MLP-MC) model, we projected the future land cover changes of Snowdonia National Park in 2050 based on historical changes in the land cover between 2005 and 2015. The land cover maps for 2005 and 2015 were acquired from “The European Space Agency CCI” global land cover product. Land cover transitions were modeled using a Multi-layer Perceptron neural network. A transition matrix was created to quantify the transition potential between the two time periods. For the sake of simplicity, we assumed that the transition probabilities (patterns of change) would remain unchanged in future and used these to predict land cover for 2050. We used a number of driver or explanatory variables to generate transition potential maps to improve the prediction accuracy of the model. These driver variables included elevation, aspect, hillshade, slope, distance to roads, distance to road nodes, distance to water channels, distance to hydro nodes, distance to green space sites, and distance to access points. A flow chart of the land cover and species distribution modeling is shown in Figure 1.

### Maxent model complexity and tuning

The complexity of models resultant in MAXENT environment is primarily driven by the following two factors; feature type and regularization parameter (Moreno-Amat et al., 2015). Maxent offers a range of five function forms known as “feature types” to explain the relationship between predictor variables and the probability of species occurrence. These feature types are labeled as Linear (L), Quadratic (Q), Hinge (H), Product (P), and Threshold (T) (see Phillips et al., 2004, 2006; Elith et al., 2011 for details). Maxent allows users to select and combine different function forms manually or picks functions or their combinations automatically when left in the default “Auto Feature” mode. Most of the published MAXENT-based studies rely on the default options of feature type and regularization parameters, which means that model complexity and the risk of over-fitting is completely ignored by the researchers (Muscarella et al., 2014). The second key factor that determines the complexity of MAXENT models is the regularization parameter. As part of the modeling process, MAXENT pushes or modifies the predictor values (such as variance and mean) of environmental variables as close as possible to the values describing actual presence points, which frequently leads to over-fitting of the model. To counter over-fitting, MAXENT uses the regularization parameter to control the complexity of models (the default value is 1). The regularization parameter limits the number of “features” in the model, depending on the number of presence records (fewer records allow for fewer features to be included). A higher value of the regularization parameter penalizes the number of features and thus leads to less complex models (Merow et al., 2013). Various studies have confirmed that calibrating MAXENT models with default settings frequently leads to highly complex models, a species-specific tuning of the model is thus recommended (Moreno-Amat et al., 2015). In this study, we generated all possible combinations of features types in combination with a range of regularization parameter values; 0.1, and then 1–10 with an increment of 1. We then used ENMeval R package to select the model with the lowest AICc (corrected Akaike Information Criterion) value which was then used as the most appropriate (least over-fitted model) out of the whole suite of models (Warren and Seifert, 2011; Muscarella et al., 2014).

### Model calibration and evaluation

We ran MAXENT (version 3.3.3a) with the default convergence threshold of 10^−6^ and with 5,000 iterations. This number of iterations was set to allow the model a reasonable scope for convergence, thus reducing the risk of over-predicting or under-predicting the model relationships. The selected model used the “Linear” and “Quadratic” feature types and the regularization parameter of 2, as indicated by the lowest AICc value. We processed 20 model replications with bootstrap resampling which randomly allocated 75% of the occurrence records to calibration and 25% to validation. We used the average of the 20 replicate models to produce habitat suitability maps under current and future scenarios. MAXENT produces continuous suitability index in its output, 10 percentile training presence threshold was employed to convert this index into binary form (suitable and unsuitable habitat; Rebelo and Jones, 2010).

AUC (Area under the receiver operating characteristic curve) was used to test the performance of the model against actual observations (Elith et al., 2006). An AUC value of 0.5 shows that the model does not predict any better than random chance, whereas a value closer to 1 indicates better performance of the model. Based on the AUC value, a conventionally used guide for ranking the model performance is: 0.5–0.6 = Failed; 0.6–0.7 = Poor; 0.7–0.8 = Fair; 0.8–0.9 = Good; 0.9–1 = Excellent (Swets, 1988). Jackknife test and percent variable contribution were used to assess the relative significance of predictor variables. Fitted response curves were used to visually investigate the relationship between individual variables and predicted index of environmental suitability of *R. ponticum*.

AUC was suggested not be sufficiently reliable for model evaluation, as an alternative, the Continuous Boyce Index (CBI) can be utilized a complementary evaluation index (Breiner et al., 2015). The Boyce index requires presence data only and measures how much model predictions differ from random distribution of observed presence across the prediction gradient. The continuous values of Boyce index vary between −1 and +1. Positive values indicate a model where predictions are consistent with the distribution of actual presence data, values close to zero mean that the model is not different from a random model and negative values indicate counter predictions (e.g., predicting no occurrence in areas where actual presence is recorded, Boyce et al., 2002; Hirzel et al., 2006).

## Results

### Model performance

**Figure 2:**
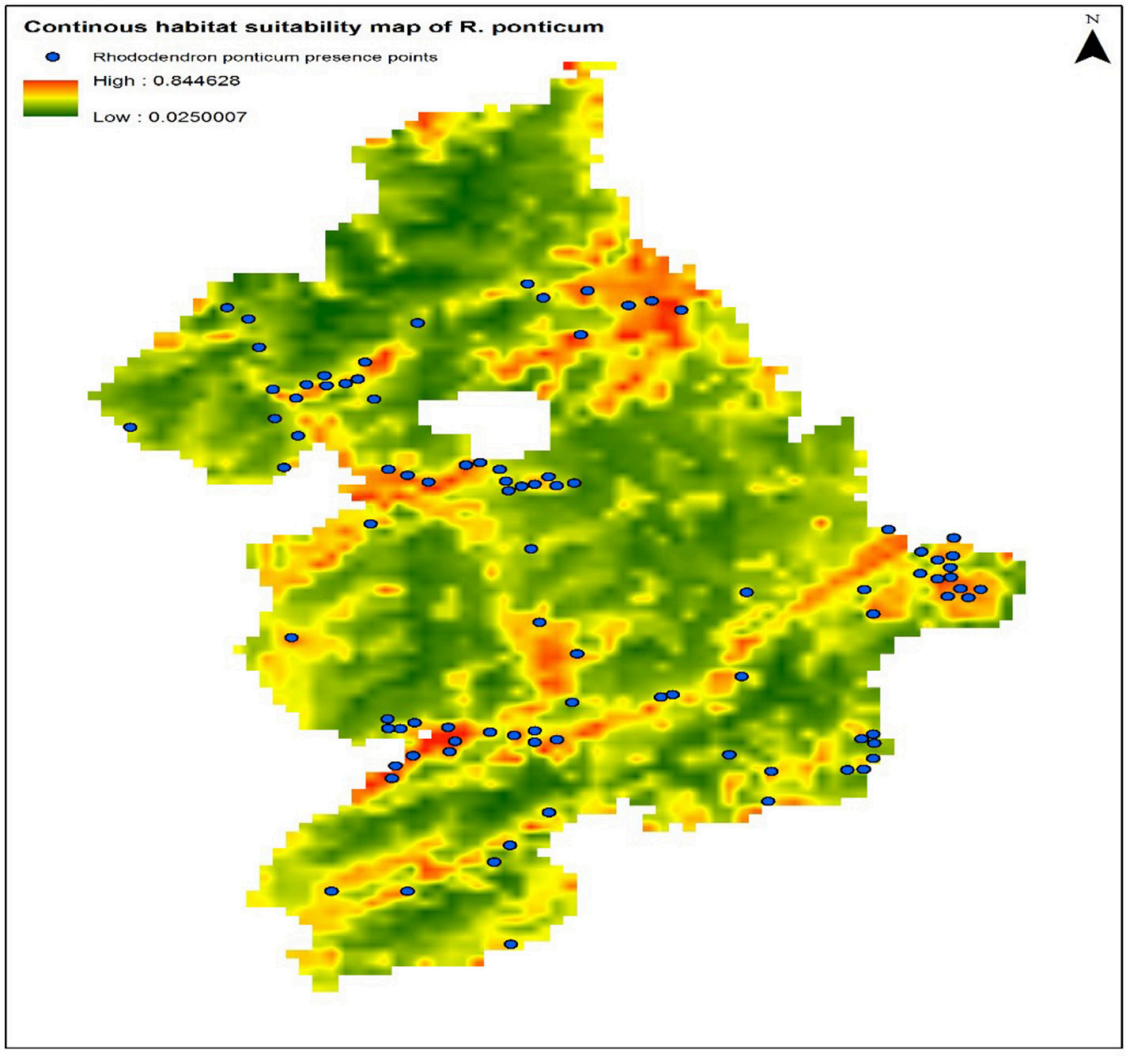
Continuous habitat suitability map of *R. ponticum* generated in Maxent model under current LCCs in Snowdonia National Park. Blue dots on the map show current distribution of species occurrence records.

The calibration test of the model specification selected on the basis of the lowest AICc showed encouraging predictive capacity: AUC_train_ = 80.0, AUC_test_ = 75.61, and CBI = 0.82. These results suggest that the predictor variables used during model calibration can predict the presence of *R. ponticum* in the Snowdonia National park with a fairly good degree of accuracy. Current distribution of *R. ponticum* on a continuous habitat suitability map for the present day LCCs is shown in Figure 2.

Comparing the predictor variables used in this model, Land Cover type contributed the most predictive power (43.3%), followed by aspect (21.5%), and altitude (15.5%, Table 2). The Jackknife test suggests that the variable which decreases the gain the most when omitted is land cover, indicating that it contains the most information absent in the other variables (Figure 1, Supplementary Data S1).

**Table 2:** Analysis of variable contribution.

**Variable**	**Percent contribution**
Land cover	43.3
Aspect	21.5
Altitude	15.5
Bio15	9.4
Bio3	4.1
Bio9	3.4
Bio2	1.6
Slope	0.9
Bio4	0.3

Close inspection of individual response curves (Supplementary Data S1) shows how the logistic prediction by a variable changes when the rest of the predictor variables are artificially kept at their average values. Starting with Land Cover, the only categorical predictor used in this study, it suggests that the presence of several land use types may have a major influence on the probability of *R. ponticum* occurrence in Snowdonia National park. The likelihood of presence is the highest in Land Cover type “8” (Mosaic tree and shrub), followed by Land Cover type “6” (Needle leaved forest). Aspect was found to be an efficient predictor of *R. ponticum* distribution, indicating that the probability of occurrence is the highest in Northern Aspect (azimuth values ranging from 337.5 to 360°). The response curve of Altitude shows that the probability of presence is negatively correlated with this variable as increasing altitude suggests a gradual decrease in the probability of species occurrence. Precipitation seasonality (BIO 15) was shown to be negatively correlated to the probability of the presence of *R. ponticum*; the species is not likely to tolerate higher seasonal variability in precipitation in Wales. It is noteworthy that the probability of species occurrence decreases from 67 to as low as 27 within a narrow band defined by 22 and 25 mm of precipitation seasonality. Response curve of BIO 9 (Mean Temperature of the Driest Quarter) shows a similar trend, *R. ponticum* probability of occurrence decreases as the mean temperature of the driest quarter increases. BIO 2 (Mean Diurnal Range) and BIO 3 are only two climatic variables which appear to be positively correlated with the probability of *R. ponticum* occurrence. BIO 4 (the coefficient of variation of the mean of monthly temperatures, represents the seasonal variation in temperature) and Slope contributed the least to the model. Response curves of both these variables suggest that probability of species occurrence would decrease with increasing values of these variables.

Our land cover change simulation of Snowdonia National Park for the year 2050 revealed that broadleaved deciduous trees, needleleaved evergreen trees and grasslands may experience a contraction in their extent, while the area under herbaceous cover, mosaic tree and shrub, mosaic herbaceous cover and shrub, or herbaceous cover may increase (Table 3).

**Table 3:** Change in area (sq. km) under the 16 land cover classes of Snowdonia National Park between current (2015) and projected (2050) maps.

**Class ID**	**Land use class**	**2015 (km^2^)**	**2050 (km^2^)**	**Change (%)**
1	Cropland	0.5586	0.5586	0
2	Herbaceous cover	3.72	4.9	+28.7
3	Mosaic cropland	8.19	8.19	0
4	Mosaic natural vegetation	6.08	6.08	0
5	Broadleaved Deciduous Trees	19.61	12.53	−36.1
6	Needleleaved Evergreen Trees	229.64	223.62	−2.62
7	Needleleaved Deciduous Trees	0.3724	0.3724	0
8	Mosaic tree and shrub	141.44	147.84	+4.52
9	Mosaic herbaceous cover	627.55	637.98	+1.66
10	Grassland	930.37	925.28	−0.54
11	Sparse vegetation	85.15	85.15	0
12	Shrub or herbaceous cover	25.75	25.94	+0.73
13	Urban areas	2.85	2.85	0
14	Bare areas	10.42	10.42	0
15	Unconsolidated bare areas	1.55	1.55	0
16	Water bodies	32.27	32.27	0

### Habitat suitability under current and future land use and climate change scenarios

Binary maps of predicted distribution of *R. ponticum* in Snowdonia National park under current and future LCCs are shown in Supplementary Data S2. Based on the output of our model, nearly 50% of the total area of the park (1,050 of 2,132 km^2^) is currently suitable for *R. ponticum* invasion. Looking into the future, the extent of habitat suitable for *R. ponticum* in Snowdonia National park is likely to be negatively affected by land cover and climate change under all considered scenarios (Table 4).

**Table 4:** Variation in suitable area (in %) for *R. ponticum* in Snowdonia National Park for current time with those identified in land cover and six future climate change scenarios for 2050 at two Representative Concentration Pathways (4.5 and 8.5).

**GCM's**	**RCP 4.5 (%)**	**RCP 8.5 (%)**
BCC-CSM1-1	−39.23	−31.84
CCSM4	−10.73	−19.13
GISS-E2-R	−35.67	−44.07
HadGEM2-ES	−8.39	−7.97
MIROC5	−3.45	−12.91
MPI–ESM-LR	−40.13	−46.78

**Figure 3:**
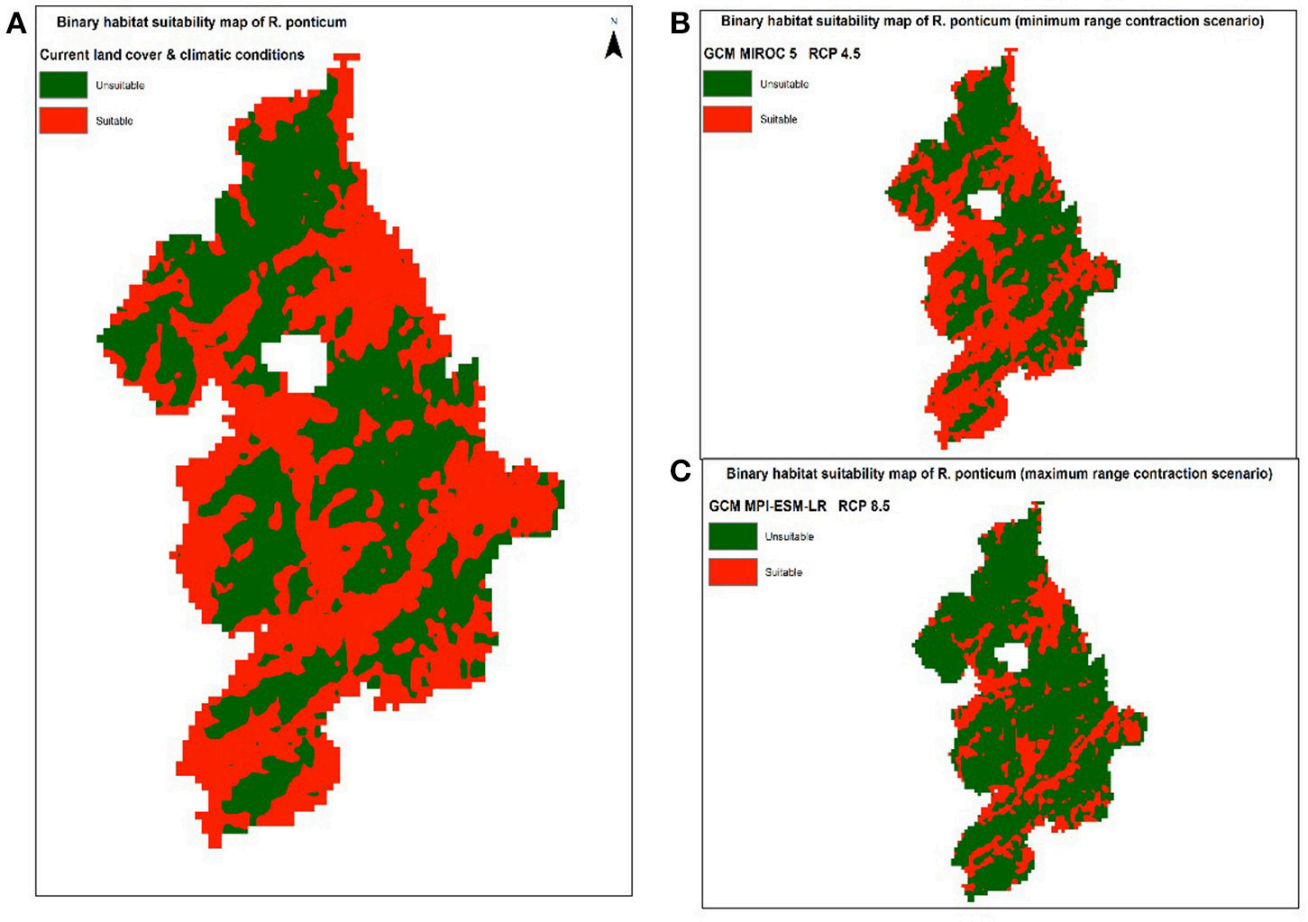
Comparison of suitable habitat range of *R. ponticum* in Snowdonia National Park under current LCCs with the minimum and maximum range contraction scenarios in future LCCs. **(A)** Binary habitat suitability map of *R. ponticum* (current land cover and climatic conditions). **(B)** Binary habitat suitability map of *R. ponticum* (minimum range contraction scenario) GCM MIROC 5 RCP 4.5. **(C)** Binary habitat suitability map of *R. ponticum* (maximum range contraction scenario) GCM MPI-ESM-LR RCP 8.5.

Under RCP 4.5, minimum contraction (−3.45%) is predicted under MIROC5 while maximum contraction (−40.13%) in suitable area may take place under MPI-ESM-LR. Under RCP 8.5, minimum (−7.97%) and maximum (−46.78%) reduction in suitability range for *R. ponticum* may be expected under GCMs HadGEM2-ES and MPI-ESM-LR, respectively. A comparison of the current habitat suitability with the minimum and maximum future range contraction (binary maps) is shown in Figure 3. Results indicate that most of the northern, northeastern and central areas of the national park are likely to become unsuitable for *R. ponticum* by 2050 (in case of maximum contraction under GCM MPI-ESM, RCP 8.5). Detailed habitat suitability maps of all future LCCs are presented in Supplementary Data S2.

## Discussion

This study presents the first attempt to delineate current distribution and investigate the impacts of changing landscape and climate on future distribution of *R. ponticum* in Snowdonia National Park. Both current and future distributions of this invasive plant are governed by an interaction of a range of factors. In the case of *R. ponticum* in Snowdonia, land cover and topography have been shown as the most influential, complemented by a range of climatic factors.

Land use has repeatedly been shown to be the key predictor variable determining plant species distribution (Yang et al., 2013). *R. ponticum* can invade a range of land cover categories, including natural to semi-natural, upland heaths, and occasionally grasslands. In Britain, earlier studies reporting on its occurrence suggest that woodland is the land cover type most affected by the invasion of *R. ponticum* (Dehnen-Schmutz et al., 2004). Our findings are in agreement with these reports; *R. ponticum* has the highest probability of occurrence in land cover categories representing “6: Mosaic Tree & Shrub” and “8: Needle Leaved Forest.” There are numerous reasons why *R. ponticum* favors woodland in Wales, for example, the availability of a microenvironment suitable for seed germination (Stephenson et al., 2006) or growing under tree canopies to spread “under-cover” and thus avoid eradiation likely play a role. Crucially, the presence of dead plant material or moss cover may be critical to *R. ponticum* establishment (Cross, 1981). In our study, Mosaic Tree & Shrub and Forests were the land cover categories which are likely to contain these substrates in the understory. Both of these land cover categories favored by *R. ponticum* are predicted to experience only a minor change (a decrease of −2.62% in category “6” while an increase of 4.52% in category “8”). Thus, the range contraction in *R. ponticum* seems to be much larger than the predicted change of suitable habitat types. This suggests that the predicted contraction in *R. ponticum* future range may not be primarily governed by land cover changes. These results are in agreement with some earlier studies suggesting that species' range may drastically contract even if there is only a little shift in land cover types (Charbonnel et al., 2016). Among topographic variables, aspect makes a major contribution in our model. We show that *R. ponticum* clearly favors the northern aspect for its establishment and growth. North-facing slopes at the latitude of Wales are likely to offer greater soil moisture, in addition to lower direct insulation intensity. Many other studies on *R. ponticum, R. simsii*, and *R. ferrugineum* suggest that northern slopes (in the Northern hemisphere) offer more favorable conditions for Rhododendron growth (Taylor et al., 2013; Christiaens et al., 2014; Francon et al., 2017). Our results show that the probability of occurrence of *R. ponticum* in Snowdonia is negatively correlated with slope. Earlier studies have suggested that shallow-slope areas are typically those with high soil moisture and nutrient availability, thus offering more favorable microenvironment for plant proliferation (Kang et al., 2016). Altitude explained a minor share of the variation in the training set of occurrence observations in this study. Even though altitude is considered an indirect variable since it has no direct effect on plant growth and physiology, it acts as a very good proxy of other un-measured or un-used variables. The reported altitudinal range of *Rhododendron* in Snowdonia National Park is well within the global range inhabited by this species. Therefore, it is likely that altitude *per se* does not represent a set of critically limiting variables in our study, but more likely acts as a proxy for auxiliary variables such as hydrology, exposure to light, wind speed, soil type and others which are not included in our model. There is strong evidence that the inclusion of indirect variables can enhance the predictive performance of SDMs, however their collinearity with direct variables must be addressed (Austin, 2002; West et al., 2016).

For climatic variables, our results indicate that both temperature- and precipitation-related variables make significant contribution to model prediction, which is in agreement with earlier studies which posit that the future distribution of *R. ponticum* in Wales may be affected by climatic predictors (Kang et al., 2016). Under all GCMs considered here, habitat suitability range decreases from the current situation. Global mean temperatures may increase by as much as 4°C by the end of next century (IPCC, 2014). Increasing temperature and changes in precipitation are likely to impact species distribution (Bezeng et al., 2017), however, existing investigations paint a mixed picture; plant species may experience an increase or a decrease of their current range (Thomas et al., 2004; Bradley et al., 2010). A study investigating potential changes in the future distribution of a 100 of the world's worst invasive species concluded that potential range of the majority of these species would increase (Bellard et al., 2013). Contrary to this, there is evidence of a range reduction of over 80 invasive species in South Africa under varying climate change scenarios (Bezeng et al., 2017). Similarly, many other ecological modeling studies have reported a possible contraction in suitable habitat of different species (Smale and Wernberg, 2013). There are studies even predicting a complete loss of species' suitable habitat (Midgley et al., 2002; Bomhard et al., 2005; Sarmento Cabral et al., 2013). Detailed studies are thus required to investigate how an existing plant invasion will be modified by changing climatic conditions; it is not likely that all invasive species will benefit from new conditions.

The fact that *R. ponticum* is an alien invasive species in the area under consideration is an important aspect of this study. Invasion is a dynamic process guided by an inherited set of traits and environmental conditions (Erfmeier and Bruelheide, 2004). One of the ways to build a species distribution model is to use climatic data and occurrence records from the native range of the invasive species under consideration and to project it to the invaded region (Kaplan et al., 2012). However, we argue that this approach may yield a poorly performing model due to the mismatch between key environmental variables between native and invaded regions. This argument is borne out by the notion that invasives are a good example of species with a potential to expand their range beyond the climatic envelope defined in their native range (Rödder and Lötters, 2009). A number of studies have confirmed this idea by concluding that invaded locations cannot necessarily be predicted from native distribution records of invasive species (Fernández and Hamilton, 2015). If the goal is to evaluate range expansion of invasive species then it could be useful to fit the model with data from native range (Araújo and Guisan, 2006), but when building models to predict changes in the invaded area under climate change scenarios, it may be much more useful to use data describing affected location (Jeschke and Strayer, 2008).

### Recommendations for future studies

Given that 14 out of 19 climatic variables originally considered for this study were excluded due to high correlation with variables chosen for the best performing model, an in-depth analysis of the sensitivity of *R. ponticum* distribution to the remaining variables may reveal interesting insights. We made use of only six GCMs and two RCPs scenarios for the sake of simplicity, but further studies including more numerous GCMs and RCPs may prove useful for improved prediction of future distribution and a better understanding of the sensitivity of *R. ponticum* to climate change. In line with the consideration of native vs. invaded climate envelope, further studies should compare model performance based on training on native and invaded climatic envelope range. Distribution models may be improved by the inclusion of high resolution variables derived from remote sensing and lidar (canopy height, cover, vertical distribution ratio etc.), variables such as vegetation density or stand height have been shown to significantly improve SDMs (Yang et al., 2013; Ackers et al., 2015). In this study, the land cover variable is considered as a proxy for the soil properties (Davis et al., 2014). For example, *R. ponticum* is known to grow under semi-shade on moist, loamy soils. Thus, the land cover types “Forest” “Bog & Mosses” & “Herbaceous cover” can be thought to act as proxy for these soil types while land cover types such as “open fields,” “bare land”, “urban areas,” and “rocks” can be considered the areas where soils types are the least favorable for this species. Results of this study confirm these observations. We however, recommend incorporating soil variables for future studies to further improve the accuracy of the model.

In this study, we projected land cover changes from 2015 to 2050 based on the land cover transition potential between 2005 and 2015. This is a simplistic and frequently adopted, “business-as-usual” approach of land use change modeling, which however may not be realistic. We suggest that the impact of contrasting socio-economic scenarios on likely future land use should be included to achieve a more representative prediction of future distribution.

## Conclusions

This study presents the results of correlative ecological modeling exercise based on an assumption that land cover and climatic variables have a dominant role in current and future distribution of *R. ponticum* and that the ecological niche for this species remains conserved across time. We show that, contrary to expectation, future distribution range of this species in Snowdonia National Park may decrease as a result of projected climate and land use changes. An extension of this modeling approach to the entire landscape of UK might help to understand the combined effects of these predictor variables to future distribution of *R. ponticum* across the country.

## Data availability

Rhododendron presence records used in the study can be accessed from COFNOD (http://www.cofnod.org.uk/Home).

## Author contributions

SM, GG, and ML conceived the ideas; SM and KI collected the data; SM, GG, ML, and KI analyzed the data; SM, ML, and GG wrote the manuscript.

### Conflict of interest statement

The authors declare that the research was conducted in the absence of any commercial or financial relationships that could be construed as a potential conflict of interest.
