# Supplementary material for: Land Cover and Climate Change May Limit Invasiveness of Rhododendron ponticum in Wales
Source: Front Plant Sci. 2018 May 18;9:664. doi: 10.3389/fpls.2018.00664 (PMC5968121; doi:10.3389/fpls.2018.00664)
Supplement: Supplementary file 1 [file Image_1.PDF]

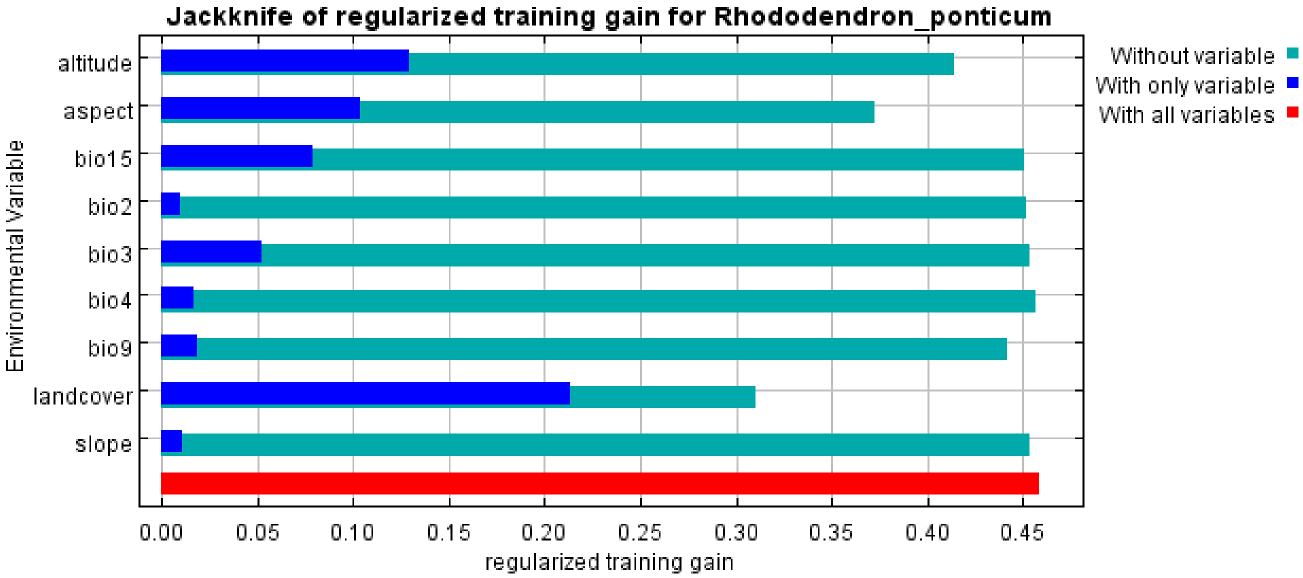

Fig 1. Jackknife of regularized training gain for presence of *Rhododendron ponticum* as predicted by the set of 9 selected predictor variables.

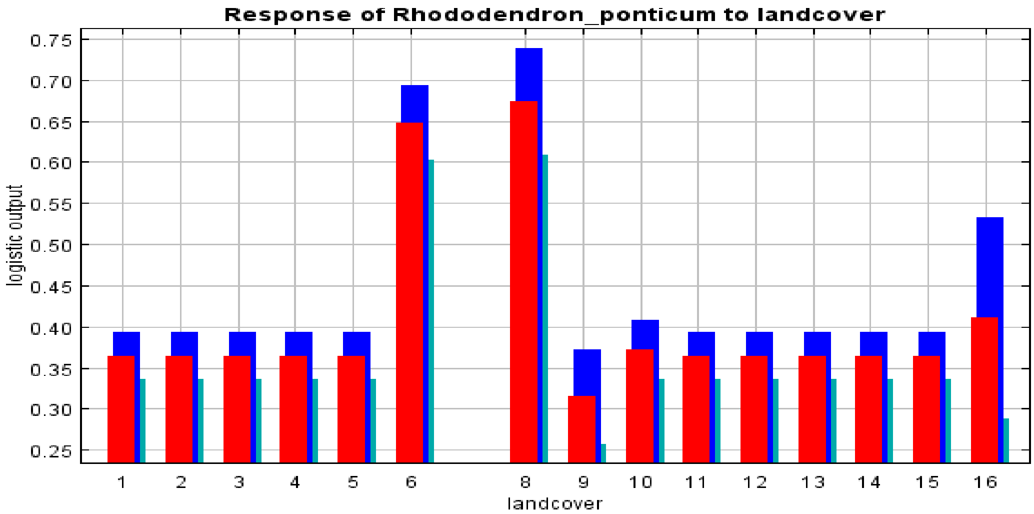

Fig 2. Probability of presence of *Rhododendron ponticum* in Snowdonia according to the land cover type. Red bars indicate mean response of 100 replicates while blue bars denote standard deviation. X-axis legend: (1) Cropland, rainfed. (2) Herbaceous cover. (3) Mosaic cropland (>50%) / natural vegetation (tree, shrub, herbaceous cover) (<50%). (4) Mosaic natural vegetation (tree, shrub, herbaceous cover) (>50%) / cropland (<50%). (5) Tree cover, broadleaved, deciduous, closed to open (>15%). (6) Tree cover, needleleaved, evergreen, closed to open (>15%). (7) Mosaic tree and shrub. (>50%) / herbaceous cover (<50%). (8) Mosaic herbaceous cover. (>50%) / tree and shrub. (<50%) (9) Shrubland. (10) Grassland. (11) Lichens and mosses. (12) Sparse vegetation (tree, shrub, herbaceous cover) (<15%). (13) Shrub or herbaceous cover, flooded, fresh/saline/brakish water. (14) Urban areas. (15) Bare areas. (16) Water bodies

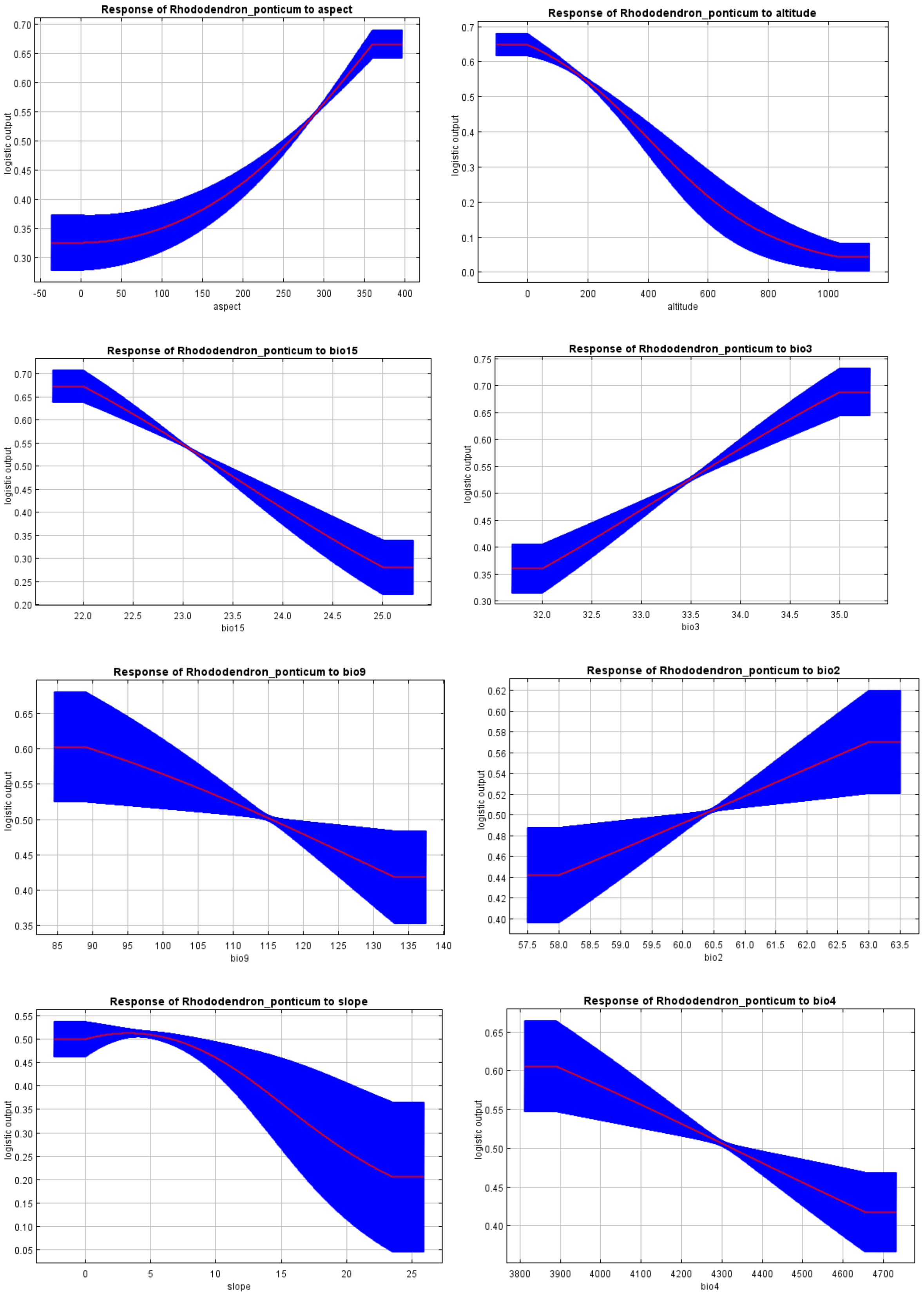

Fig 3. Probability of presence of *Rhododendron ponticum* in Snowdonia Natioanl Park, as influenced by A) aspect (° azimuth), B) altitude (m.a.s.l), C) precipitation seasonality (BIO15, mm), D) isothermality (BIO3), E) mean temperature of the driest quarter (BIO9, °C), F) mean diurnal range (BIO2, °C), F) G) slope (°) and H) E) coefficient of variation of mean of monthly temperatures (BIO4)
