# Supplementary material for: Land Cover and Climate Change May Limit Invasiveness of Rhododendron ponticum in Wales
Source: Front Plant Sci. 2018 May 18;9:664. doi: 10.3389/fpls.2018.00664 (PMC5968121; doi:10.3389/fpls.2018.00664)
Supplement: Supplementary file 2 [file Image_2.pdf]

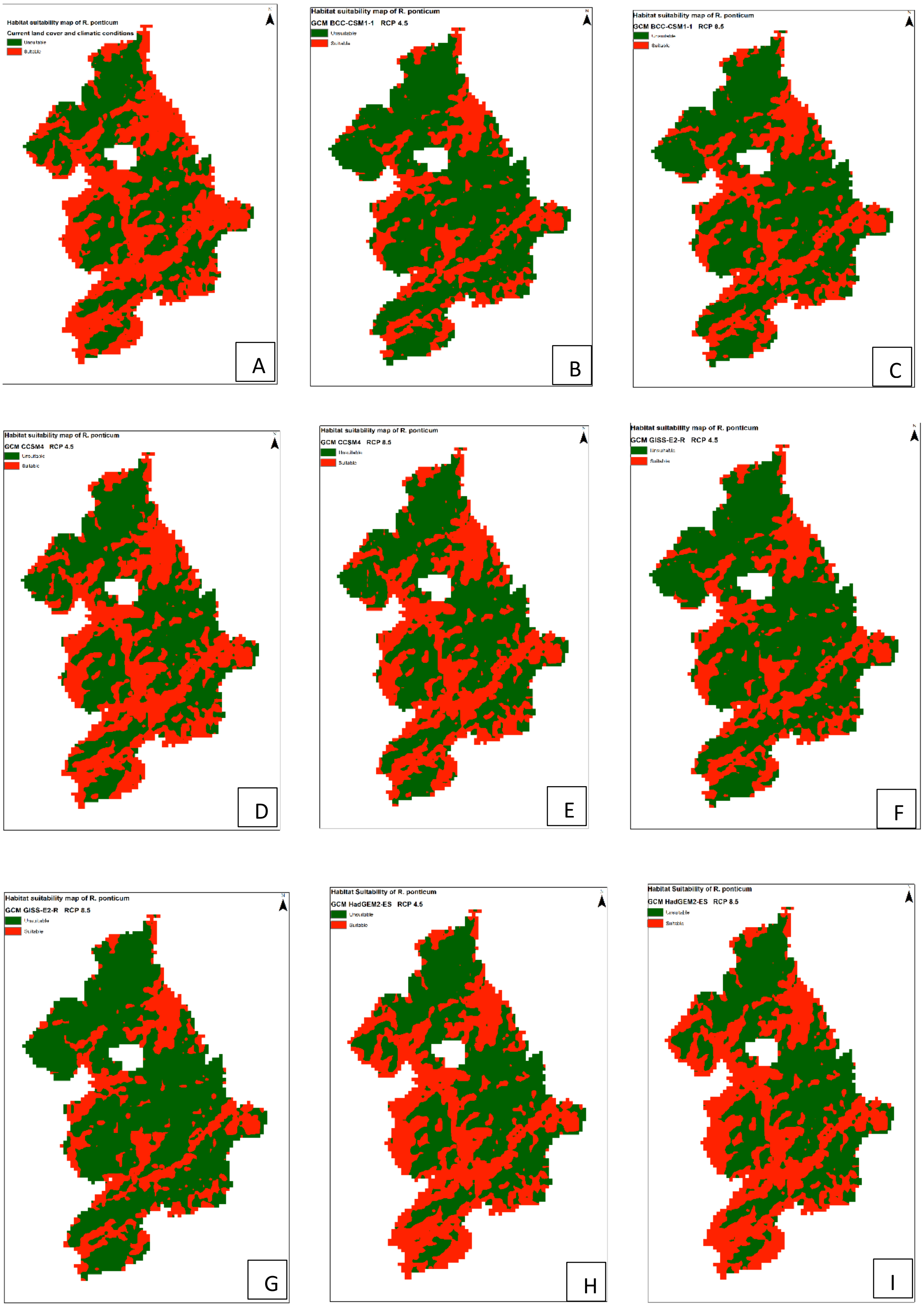
Habitat suitability map of R. ponticum
Current land cover and climatic conditions
A
Habitat suitability map of R. ponticum
GCM BCC-CSM1-1 RCP 4.5
B
Habitat suitability map of R. ponticum
GCM BCC-CSM1-1 RCP 8.5
C
Habitat suitability map of R. ponticum
GCM CCSM4 RCP 4.5
D
Habitat suitability map of R. ponticum
GCM CCSM4 RCP 8.5
E
Habitat suitability map of R. ponticum
GCM GISS-E2-R RCP 4.5
F
Habitat suitability map of R. ponticum
GCM GISS-E2-R RCP 8.5
G
Habitat Suitability of R. ponticum
GCM HadGEM2-ES RCP 4.5
H
Habitat Suitability of R. ponticum
GCM HadGEM2-ES RCP 8.5
I

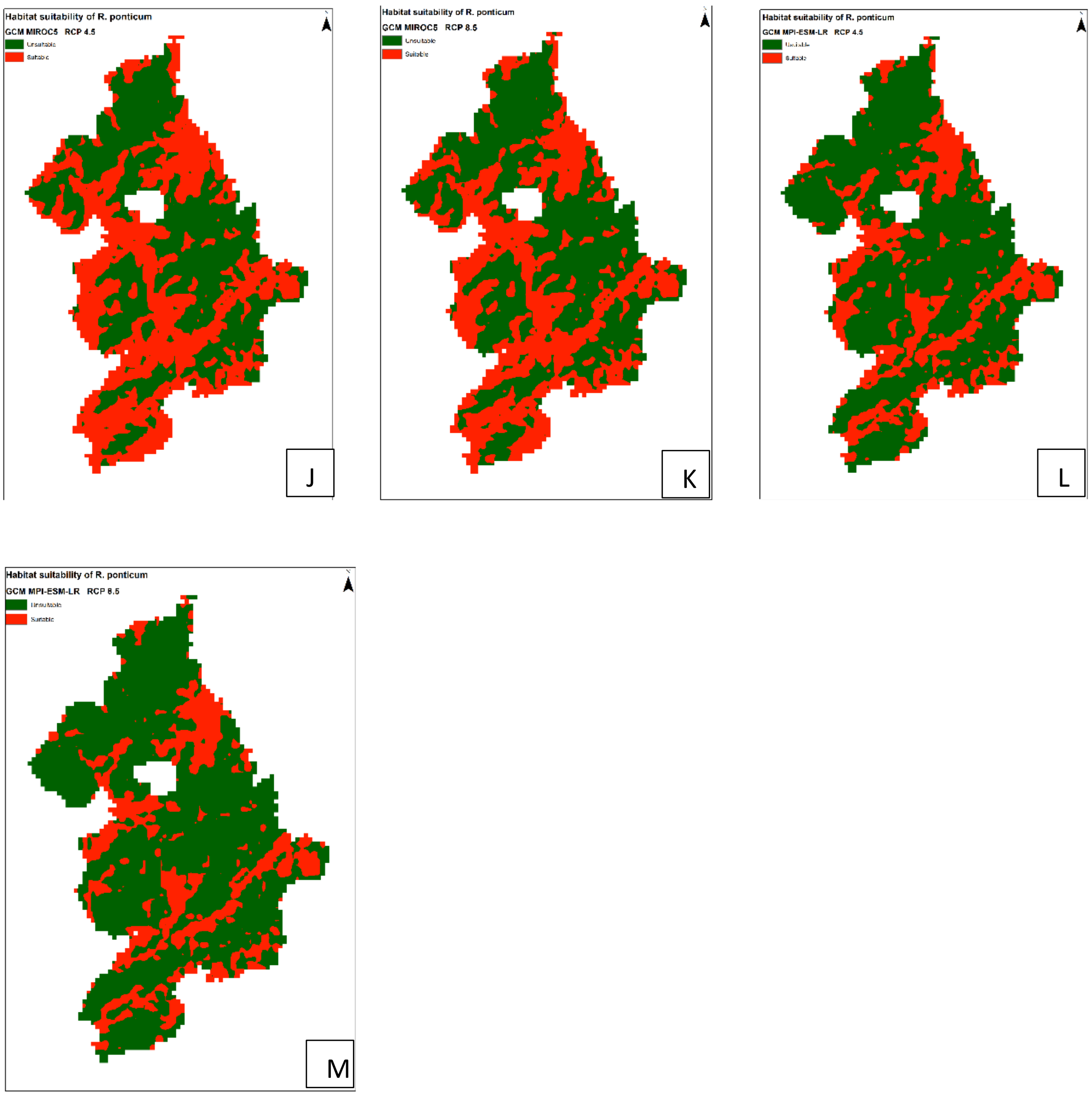

Fig. 4. Binary habitat suitability maps of *R. ponticum* in Snowdonia National Park under current land cover and climatic conditions (A) and future land cover and climate change scenarios: BCC-CSM1-1 (B), BCC-CSM1-1 (C), CCSM4 (D), CCSM4 (E), GISS-E2-R (F), GISS-E2-R (G), HadGEM2-ES (H), HadGEM2-ES (I), MIROC5 (J), MIROC5 (K), MPI-ESM-LR (L), MPI-ESM-LR (M)
